# Supplementary material for: What matters most to patients with severe aortic stenosis when choosing treatment? Framing the conversation for shared decision making
Source: PLoS One. 2022 Aug 11;17(8):e0270209. doi: 10.1371/journal.pone.0270209 (PMC9371337; doi:10.1371/journal.pone.0270209)
Supplement: S1 Appendix — (DOCX) [file pone.0270209.s001.docx]

# Supporting Materials

**Contents**

[Supporting Materials 1](#_Toc89196406)

[Supporting Methods 2](#_Toc89196407)

[Detailed Recruitment 2](#_Toc89196408)

[Detailed Study Procedures 2](#_Toc89196409)

[Detailed Statistical Analyses 3](#_Toc89196410)

[Supporting Figure 1. Prioritized Treatment Goals; n=43 5](#_Toc89196411)

[S Figure 1 Legend 5](#_Toc89196412)

[Supporting Figure 2. Prioritized Treatment Features, n=41 6](#_Toc89196413)

[S Figure 2 Legend 6](#_Toc89196414)

[Supporting Figure 3. Dendrogram of Treatment Goals 7](#_Toc89196415)

[Supporting Figure 4. Dendrogram of Treatment Features 8](#_Toc89196416)

[Supporting Legend for S Fig 3 and S Fig 4. 9](#_Toc89196417)

[References 10](#_Toc89196418)

## Supporting Methods

## Detailed Recruitment

To identify health care provider (HCP) advisers and patient advisers, we used a purposive to convenience recruitment strategy. To identify study participants, we used a purposive strategy. For patients, we targeted diversity in age, treatment chosen, complications, time since treatment, geography, and race/ethnicity. For referring providers, we targeted diversity in age, gender, and race, and type of cardiology practitioner. Referring HCPs included interventional cardiologists, a cardiac surgeon, general cardiologists, and a nurse practitioner. Participants provided informed consent online.

Patient recruitment through HCPs was done through ads, flyers, email invitations and direct contact with their patients. Patient recruitment through patient advisers included personal referrals. Study ads provided ways to access to the intake screener (QR code or email) where participants could opt-in, undergo online screening for eligibility, give informed consent and complete the baseline survey. Participants were invited to participate between January 2020 and December 2020.

We cannot quantify how many study invitations were distributed to potentially eligible participants. Only those who were interested in participating either responded to the email or provided their email address. 11 people started but did not complete the screening component of the intake survey. An additional 18 people completed the intake survey but did not respond to subsequent invitations to participate in specific surveys, hence did not provide informed consent. Some of the reasons include illness, loss of interest, limited internet literacy, and timing (responding after activities were completed).

Informed consent was obtained online for each study activity. This study was approved by the New England Independent Review Board.

## Detailed Study Procedures

To increase geographic and racial diversity, we offered both online and in-person activities. In-person activities were conducted in handicap-accessible facilities in Lebanon, NH. During the COVID-19 pandemic, all activities were conducted online. All online surveys used customized Qualtrics© software.

We used a multi-step cognitive mapping approach in which Nominal Group Technique (NGT) meetings were used to elicit and prioritize patients’ treatment goals and treatment features.(1-7) Subsequent card sorting exercises, involving a larger sample of patients, rated and clustered the prioritized goals and treatment features. Multidimensional Scaling (MDS) and Hierarchical Cluster Analysis (HCA) were used to analyze card sort responses. (8-14) NGT participants could elect to participate in subsequent card sorting activities.

Five to nine participants were assigned to each NGT group. Groups were facilitated by an experienced moderator (NC). In addition to the moderator, two other people were present in the 3 in-person meetings**.** CF and SD assisted with logistics. The interviewer (NC) and non-participants in the room for in-person NGTs (CF, NC) were female. NC has extensive training and experience with the methods used.(15-17) Participants were aware that NC was a physician and researcher in the field of shared decision making. NC acknowledged being a primary care provider but not a cardiologist and that she no longer practices clinical medicine. Neither NC nor CF had a relationship with any of the study participants.

Each in-person NGT meeting lasted approximately 90 minutes, including a break. Refreshments were provided before and after the in-person meetings. To standardize meetings across sites, instructions were provided through a series of instructional videos. Participants were asked to silently respond to one carefully worded question (pre-tested with patient advisers), writing down as many brief responses as they wished. To elicit treatment goals, patients were provided brief background information and asked, “*What are the specific goals that you think are most important to consider when deciding about treating aortic stenosis?”.* Similarly, to elicit treatment features, participants were asked: “*What specific features, characteristics, or questions about treatment are most important to you when thinking about treating aortic valve stenosis*?” Participants could submit sensitive topics anonymously. Next, each subject read aloud one of their responses in a round-robin until all unique responses were recorded on a shared list. The moderator read anonymous topics. Each item on the list was reviewed by the group for clarity and redundancy. Finally, participants were asked to silently identify and rank the nine most important items (the maximum number that can be effectively ranked). (18, 19) Within each NGT meeting, weights were assigned to each ranked item (i.e., 9 points for the 1st, 1 point for the 9th) and participant scores were summed to develop an aggregated, prioritized list. ^20^

A 2-step online NGT protocol mirrored the in-person protocol and was previously validated.^15^ Participants in each NGT group participated asynchronously. The instructional videos used in in-person meetings explained procedures. In step one, participants read the NGT question and submitted their responses online. These responses were consolidated independently by two facilitators (NC, CF), with patient advisors resolving any differences. A cardiologist on the research team (MC) provided clarification of some of the terms to facilitate interpretation. In step two, participants were shown the consolidated list, asked to evaluate items for clarity and redundancy and to select and rank the top 9 items.

The data collected during the NGT meetings were the preference items hand-written (or entered online) by each participant and the rankings that each participant assigned to their top 9 items during the meeting. We did not use field notes or audio/visual recording for data collection.

We conducted NGTs until saturation was achieved.

After all of the NGTs were completed (in-person and online), two consolidated lists were generated (one for treatment goals, one for treatment features) by carrying forward all ranked items from the NGTs conducted on that topic, removing or rewording confusing items, and consolidating items that conveyed the same meaning. Patient advisers guided this process. An HCP adviser (MC) was asked to clarify some items to facilitate this process.

The consolidated lists of treatment goals and treatment features were used for online rating and card sorting exercises (conducted separately). Participants rated the importance of each item using a 5-point Likert scale (*very important* to *not important*). Participants were then asked to sort the items into 3 to 13 groups (“how you see these items going together?”), and then to create a name or label for each group.

## Detailed Statistical Analyses

Using customized software, card sort data for goals and features were separately transformed into a co-occurrence matrix according to how often two items were sorted into the same group. MDS (‘PROXCAL’ software algorithm) was applied to the group co-occurrence matrix using Euclidean (i.e., straight-line) distances. MDS maps the spatial relationships between the items (either the treatment goals or treatment features) based on the perceived similarity of items. The more often that items were sorted into the same group, the closer together they appear in the map. (20, 21) The space can be multi-dimensional, but only 2-dimension solutions are readily interpretable. The axes labels were labeled as the Timeline (making a decision to recovery), and Internal factors to external factors. These labels were created by the investigators (NC and CF) by examining the arrangement of items along each axis. The process started by contrasting items at the extremes of each axis, drawing upon the cluster labels created by patients. We evaluated the goodness-of-fit using the stress statistic, which indicates the differences between the observed and modeled data.(20) Values < 0.15 indicate close correspondence between modeled and observed data.(22)

Hierarchical cluster analysis (HCA)(23) yielded a dendrogram tree whose branches depict possible clusters. Patient advisers then visually inspected the dendrogram and accompanying list of items to compose clusters and provide labels for each cluster. This was done during videoconferencing calls where the investigator (NC) shared her computer screen with the patient to explain the dendrogram and side-by-side list of items. Serial rounds of review of the cluster composition and labels occurred, drawing in more patient advisers as needed. The process continued until no further changes were suggested. Analyses used IBM SPSS Version 26.

Heterogeneity analyses assessed the similarity between individual responses. These analyses compared all possible pairs of responses. We compared the ratings of each pair (32 goals, 46 features). For example, for the 46 treatment features, we had responses from 41 subjects. That yielded 820 possible pairs. For each pair, we performed 46 subtractions and took the absolute value of the mean of the paired difference. The absolute value was used because the direction of the differences was inconsequential, and this avoided having higher ratings on one item cancel out lower ratings on another item. We ranked pairs according to the percent of ratings that differed within each pair. We counted the number of pairs that differed from each other by more than 50% and divided this number by the total number of pairs to obtain the proportion of pairs that differed by more than half. That is the proportion of pairs that had substantially different ratings on either treatment goals or features. For 46 treatment features, we had responses from 41 subjects. This yielded 820 possible pairs. For 32 treatment goals, we had data from 43 subjects. This yielded 903 possible pairs. We performed the same procedure as for the treatment features and obtained the estimated proportion of similar pairs for treatment goals.

We compared the ratings of each pair (32 goals, 46 features), calculating the mean of the absolute value of the paired difference to estimate the proportion of items that differed among pairs.

# Supporting Figure 1. Prioritized Treatment Goals; n=43

^a^Importance ratings 4 = “Very important”, 3 = “Important”, 2 = “Neutral”, 1 = “Slightly important”, 0 = “Not important or Does not Apply”.

## S Figure 1 Legend

This figure depicts the mean ratings of patient treatment goals, ordered from most to least important (top to bottom).

# Supporting Figure 2. Prioritized Treatment Features, n=41

^a^Importance ratings 4 = “Very important”, 3 = “Important”, 2 = “Neutral”, 1 = “Slightly important”, 0 = “Not important or Does not Apply”.

## S Figure 2 Legend

This figure depicts the mean prioritized ratings of patient treatment goals, ordered from most to least important (top to bottom).

# Supporting Figure 3. Dendrogram of Treatment Goals


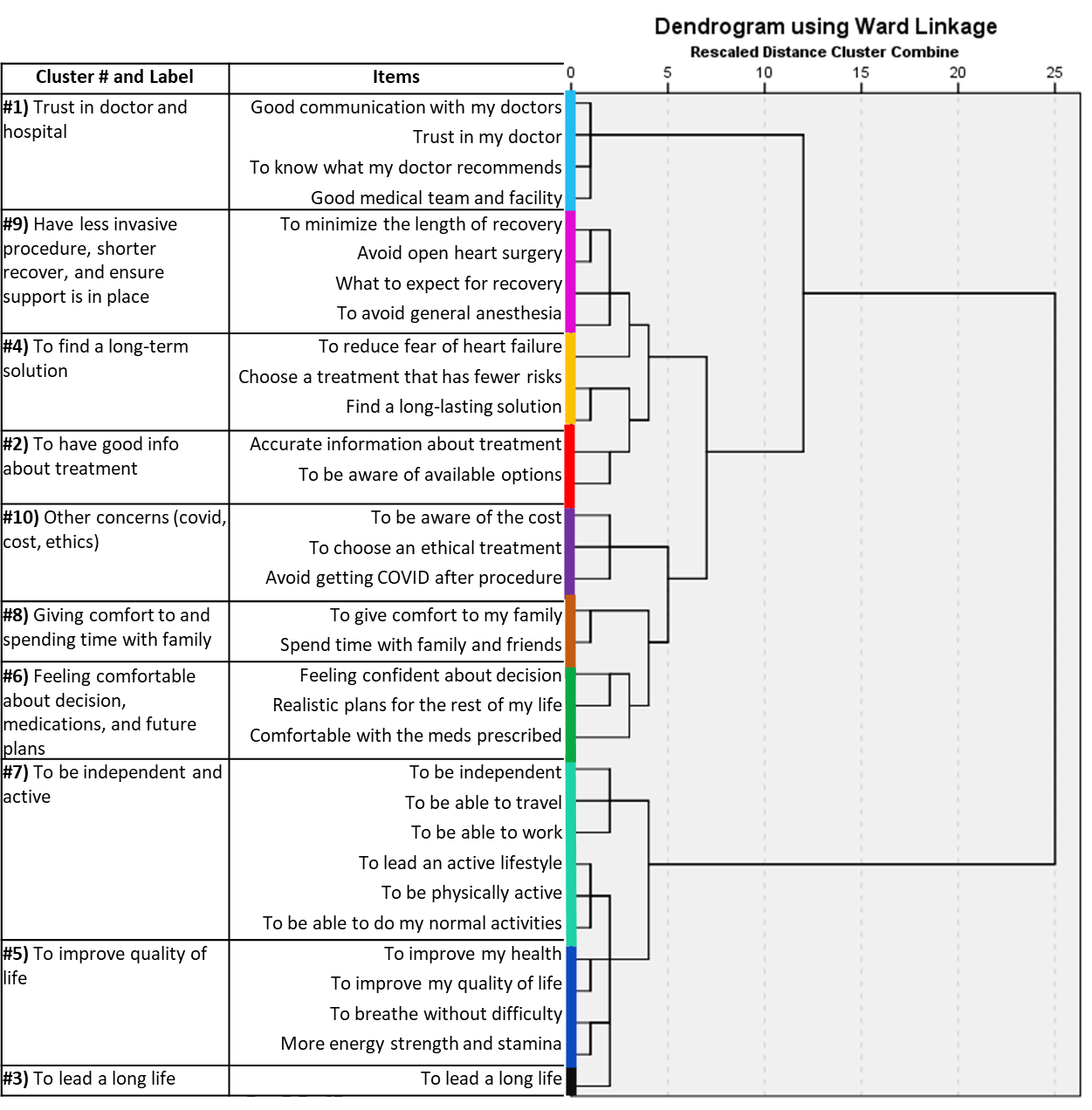


# Supp**orting Figure 4. Dendrogram of Treatment Features**


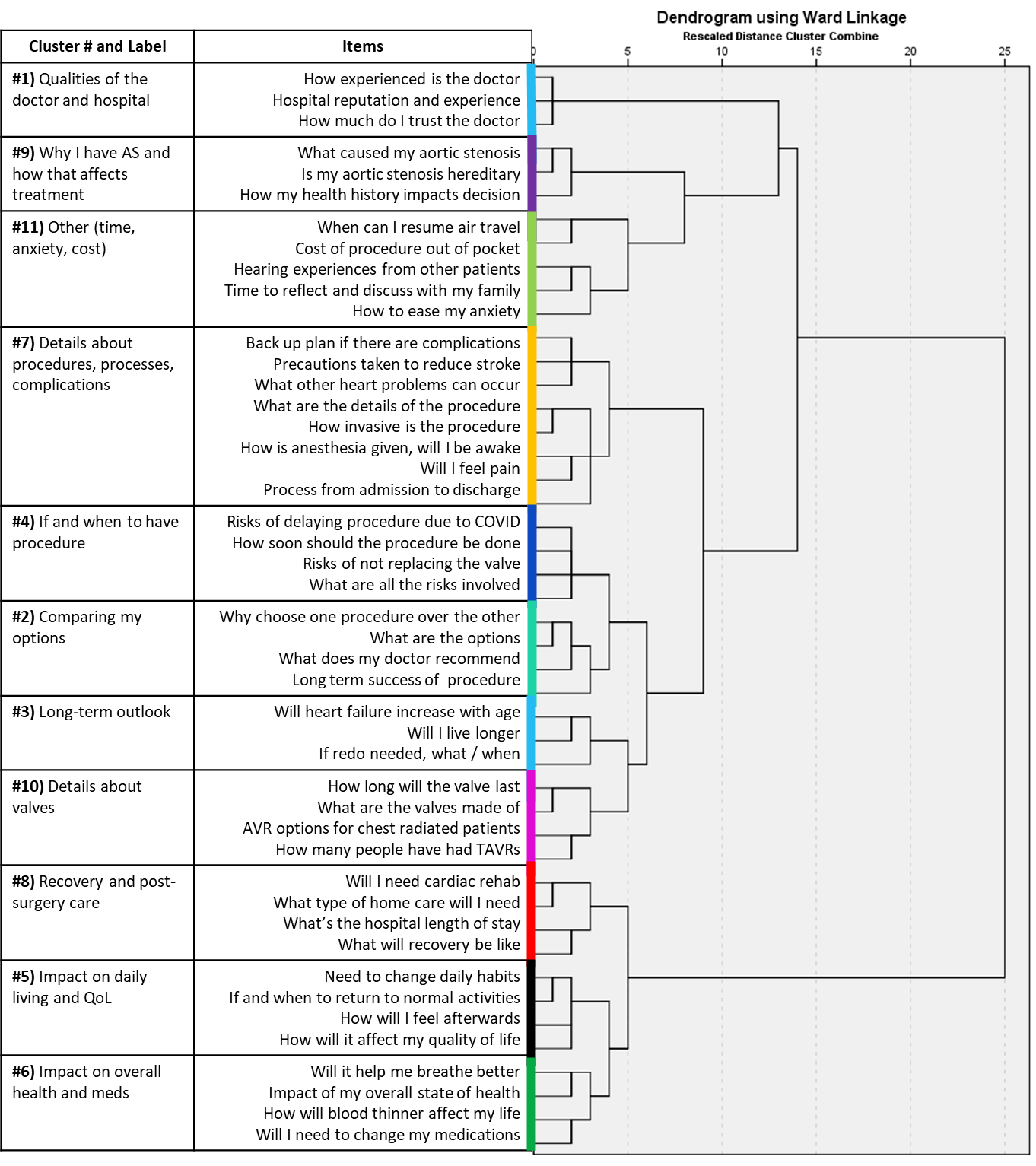


## Supporting Legend for S Fig 3 and S Fig 4.

The dendrogram tree (on the right side) was generated using hierarchical cluster analysis (HCA) of the matrix of items (treatment goals or features, respectively), which are shown adjacent to each branch of the tree. The clusters and cluster labels that were generated by participants are shown on the left. The shorter the rescaled distance of the connected branches on the horizontal axis, the more often items were sorted into the same group. Clusters are labeled by color between the dendrogram and cognitive map (Fig 2 and 3 in manuscript). Note that HCA does not specify the number of clusters, only their pattern of groupings. The cut-points for clusters were defined by patients, guided by the dendrogram tree pattern. This resulted is overlapping of some clusters, notably clusters #8, 5, and 6 in SFig 4.

# References

1. Potter M, Gordon S, Hamer P. The Nominal Group Technique: A useful consensus methodology in physiotherapy research. 2004.

2. Gallagher M, Hares T, Spencer J, Bradshaw C, Webb I. The nominal group technique: a research tool for general practice? Fam Pract. 1993;10(1):76-81.

3. Scott D, Deadrick D. The Nominal Group Technique: Applications for training needs assessment. Training & Development Journal. 1982;36(6):26-33.

4. Qu H, Shewchuk RM, Alarcón G, Fraenkel L, Leong A, Dall'Era M, et al. Mapping Perceptions of Lupus Medication Decision-Making Facilitators: The Importance of Patient Context. Arthritis Care Res (Hoboken). 2016;68(12):1787-94.

5. Qu H, Houston TK, Williams JH, Gilbert GH, Shewchuk RM. Cognitive mapping tobacco control advice for dentistry: a dental PBRN study. Am J Health Behav. 2011;35(2):228-39.

6. Malpede CZ, Greene LE, Fitzpatrick SL, Jefferson WK, Shewchuk RM, Baskin ML, et al. Racial influences associated with weight-related beliefs in African American and Caucasian women. Ethn Dis. 2007;17(1):1-5.

7. Crenshaw K, Shewchuk RM, Qu H, Staton LJ, Bigby JA, Houston TK, et al. What should we include in a cultural competence curriculum? An emerging formative evaluation process to foster curriculum development. Acad Med. 2011;86(3):333-41.

8. Fitzgerald LF, Hubert LJ. Multidimensional scaling: Some possibilities for counseling psychology. Journal of Counseling Psychology. 1987;34(4):469-80.

9. Delbecq AL, Van De Ven, Andrew H. A Group Process Model for Problem Identification and Program Planning. J Appl Behav Sci. 1971;7(4):466-92.

10. Schiffman S, Reynolds ML, Young FW. Introduction to multidimensional scaling: theory, methods, and applications. 1st ed. New York: Academic Press; 1981.

11. Kruskal JB. Multidimensional Scaling. 11th ed. Murray Hill, N.J.: SAGE; 1978.

12. Dugard P, Todman J, Staines H. Approaching multivariate analysis: A practical introduction, 2nd ed. 2nd ed. New York, NY, US: Routledge/Taylor & Francis Group; 2010.

13. Anderson RE, Tatham RL, Black WC, Hair JF. Multivariate Data Analysis (5th Edition). 5th ed. New Jersey: Prentice-Hall; 1998.

14. Aldenderfer MS, Blashfield RK. Cluster Analysis. Beverly Hills: Sage Publications; 1984.

15. Col NF, Solomon AJ, Springmann V, Ionete C, Alvarez E, Tierman B, et al. Evaluation of a Novel Preference Assessment Tool for Patients with Multiple Sclerosis. Int J MS Care. 2018;20(6):260-7.

16. Col N, Hull S, Springmann V, Ngo L, Merritt E, Gold S, et al. Improving patient-provider communication about chronic pain: development and feasibility testing of a shared decision-making tool. BMC Med Inform Decis Mak. 2020 -10-17;20(1):267.

17. Col NF, Solomon AJ, Springmann V, Garbin CP, Ionete C, Pbert L, et al. Whose Preferences Matter? A Patient-Centered Approach for Eliciting Treatment Goals. Med Decis Making. 2018 -01;38(1):44-55.

18. Delp P, Thesen A, Motiwalla J, Seshardi N. Systems tools for project planning. . Bloomington, Indiana: International Development Institute; 1977.

19. Miller GA. The magical number seven plus or minus two: some limits on our capacity for processing information. Psychol Rev. 1956 -03;63(2):81-97.

20. Kruskal JB. Multidimensional Scaling. 11th ed. Murray Hill, N.J.: SAGE; 1978.

21. Schiffman S, Reynolds ML, Young FW. Introduction to multidimensional scaling: theory, methods, and applications. 1st ed. New York: Academic Press; 1981.

22. Dugard P, Todman J, Staines H. Approaching multivariate analysis: A practical introduction, 2nd ed. 2nd ed. New York, NY, US: Routledge/Taylor &amp; Francis Group; 2010.

23. Aldenderfer MS, Blashfield RK. Cluster Analysis. Beverly Hills: Sage Publications; 1984.

24. Morris NS, MacLean CD, Chew LD, Littenberg B. The Single Item Literacy Screener: evaluation of a brief instrument to identify limited reading ability. BMC Fam Pract. 2006;7(21):21.

stylefix
