# Supplementary material for: What matters most to patients with severe aortic stenosis when choosing treatment? Framing the conversation for shared decision making
Source: PLoS One. 2022 Aug 11;17(8):e0270209. doi: 10.1371/journal.pone.0270209 (PMC9371337; doi:10.1371/journal.pone.0270209)
Supplement: Attachment — Submitted filename: Response to Reviewers.docx [file pone.0270209.s002.docx]

**Title:** What Matters Most to Patients with Severe Aortic Stenosis when Choosing Treatment? Framing the Conversation for Shared Decision Making

**Manuscript #:** PONE-D-21-38629

**Response to Reviewer Comments:**

We are grateful for the thorough review that our manuscript received and for the helpful feedback that we have used to strengthen the manuscript. We have done our best to address all the concerns raised.

**Response to Reviewer #1**

Minor comments:
**Comment 1**. Page 2, line 2: “NGT” is not defined prior to its first use.

**Response**: This oversight has been corrected. NGT stands for Nominal Group technique. (Page 2, line 2)

**Comment** 2. Page 3, line 4: “WCG” is not defined.

**Response**: This has been corrected. Western Institutional Review Board (WIRB) Copernicus Group (WCG) Independent Review Board. (Page 3, lines 6-7)

**Comment** 3. Could the authors expand a bit more on how the change to virtual groups (rather than in-person) may have affected their results?

**Response**: The change to virtual groups should not have substantially affected our results because we had previously validated the similarity between the in-person and online protocols. Because we use the same video recordings of instructions for each component of the NGT, the 2 protocols closely mirror each other.

We added a phrase to the methods section to establish the similarities between the online and in-person protocols: “An online *asynchronous* NGT protocol *that mirrored the in-person protocol* had been previously developed and validated.” (Page 4, lines 3-4).

We also added 2 sentences in the Discussion section: We combined findings from online and in-person activities, recognizing that the method of collecting responses may influence those responses. Asynchronous online activities support more inclusive sampling and thoughtful responses by removing time constraints.” (Page 18, lines 17-20)

**Comment** 4. Can the authors please specify the repository for their data and insert a statement into the methods section about its availability and location?

**Response**: We would like to make the data available through the Dryad depository, if possible. We added a statement as requested: “The data underlying our findings are available through the Dryad depository.” (Page 4, line 6.) I have never used this depository before and do not know how to access it, as it appears to be limited to members. If Dryad is not available, I can post the data through whatever mechanism is recommended.
